# Experimental Pathology

**Published:** 1860-10

**Authors:** Claude Bernard


### EXPERIMENTAL PATHOLOGY.

BY M. CLAUDE BERNARD.

In examining the various tissues which enter into the composition of the living frame, it is easily ascertained that peculiar and distinct properties are separately enjoyed by each of them; and that, in this manner, they are enabled to fulfill the various functions which devolve upon them. At the same time we discover that certain general systems exist, which, by their distribution are brought into constant communication with all the various elementary tissues of the body; you have already named the vascular and nervous systems. The blood, as you are well aware, is brought through the capillaries to every part of the body, and its presence being indispensable to the physiological existence of all living tissues, they rapidly perish when accidentally deprived of this all important and vivifying fluid. The blood, therefore, must be looked upon as the medium in which all our tissues have their being; it brings them in contact with an immense number of substances, derived from various sources, some of them drawn from the atmosphere through the process of respiration, others again introduced into the economy through various absorbant surfaces, and all of them equally necessary to the continuance of the physiological conditions of life.

Such, however, are not the only bodies which the circulating fluid brings into contact with the primitive elements of which our organs are composed; the blood is, at the same time, the vehicle through which medicinal agents, intended to modify their properties, are conveyed into living tissues, and penetrate into their substance. We must, in consequence, consider medicines as physiological agents of an unusual nature; introduced into the same channel as the bodies with which it is natually supplied, they reveal their presence exactly in the same manner, viz: by giving birth to a series of phenomena peculiar to each substance, aud widely different from those which exist in the physiological state. But another question now presents itself; *how* are these foreign substances enabled to act upon our tissues? Not through their physico-chemical properties, as has been sufficiently proved in our last lecture; a *physiological* property must, therefore, be had recourse to, as in the case of all the powers which support life. When oxygen, for instance, is brought into contact with elementary, nervous, or muscular fibres, it quickens their activity, and keeps alive all their properties within them; and, when deprived of so powerful a stimulus, both nervous and mnscular fibres quickly die. Similar actions are, no doubt, in their intimate essence, of a physical or chemical nature; but in the present state of our knowledge, the observer can only point to a physiological agency.

Histological elements, as we have already shown, are, in every case, endowed with special properties; and the wider is the difference which separates one tissue from another, the higher is the animal's rank in the scale; in the nobler species peculiar organs are adapted to each individual function; and in no other animal is their number so great as in man. We are, therefore, justified in supposing that the primitive muscular fibre, for instance, is widely different in every respect, from the nervous filament and the glandular cell; and, in consequnce, that agents entirely powerless as regards all other tissues, may be found to exercise over the muscles an almost unbounded influence.

Among the innumerable agents which possess the power of destroying life, there exist an immense number which the medical philosopher cannot, by any means, consider as diseases; no one, for instance, would think for a moment of giving that name to the bullet which slays the soldier in the battle, or the thunderbolt which strikes the plowman dead in the field; the same, of course, might be said of all the causes of violent death; now, reasoning by analogy, might we not place a large number of so-called diseases in the same class? A tumor which, by its situation, compresses the windpipe, and gradually increasing in size, superinduces suffocation at last; a stricture of the œsophagus, which prevents the passage of food into the stomach, and ultimately starves the patient to death; are not these mechanical obstructions, which, acting as impediments to some of the more important functions of life, gradually bring on death, by a slow, but inevitable process? It would, of course, be superfluous to adduce other instances to the same purpose, but we are supported by the authority of Professors Trosseau and Pidoux, in assimilating to such cases those valvular affections of the heart, which by constantly opposing the natural course of the blood, sooner or later produce a complete stasis of the circulation.

The word disease, must, therefore, be understood to signify disorders of a more extensive nature, the existence of which is revealed by phenomena of an entirely general character. Such, however, are frequently the results of local affections; the fact is established by daily experience; and we need only allude to the well-known effects of wounds and sores, in order to convince you that local causes frequently become the starting point of general diseases. The results of our physiological experiments are, in this respect, perfectly in accordance with clinical observation; let, for instance, a poison be introduced under an animal's skin; its action, during the first few moments is entirely confined to a single point, but within a given space of time, its effects are felt throughout the living frame; in what manner can similar facts be best explained? Such is the problem, the solution of which we are about to seek; the difficulty, as you perceive, has been distinctly stated.

That poisonous substances cannot act upon the tissues which compose the body, without having previously been absorbed, is perfectly clear; but when, under their influence, general phenomena are produced, the vessels or nerves must evidently have been called into play; the vascular and nervous systems alone, as you are well aware, are distributed throughout the body in one unbroken chain; and physiology, in this case, abundantly confirms the inferences drawn from our knowledge of anatomy. The well-known experiments of Magendie have shown that injecting into the arteries of a limb any given toxic substance, its ordinary effects may be indefinitely suspended, by tying the veins which connect the diseased part with the remainder of the economy. The animal's life might, in this manner, be almost indefinitely prolonged; but as soon as the vessels are untied, the poison flows into the torrent of circulation, and gives rise, as usual, to a series of phenomena which end in death. The fact is well known to the inhabitants of tropical countries, who, when bitten by venomous serpents, immediately apply a close ligature over the limb, in order to prevent the passage of the venom into the blood, until proper remedies have been applied.

But the vessels which convey into the depth of our tissues both poisonous substances and the ordinary elements of nutrition, belong, as you are perfectly aware, to the arterial system; on the other hand, all foreign bodies which pass into the blood through the process of absorption, are conveyed into the arterial circulation by the veins; we thus thus discover that when general symptoms have been produced in this manner, the two great divisions of the vascular system have both been called into play. It sometimes occurs, however, that the noxious principle which circulates in the veins is eliminated during its passage; such, for instance, is the case with sulphuretted hydrogen, one of the most dangerous substances we are acquainted with; when injected into the veins, it escapes from the lungs, and is, in this manner, prevented from passing into the arterial current; and the animal, in consequence, does not experience the slightest injury from the operation, the poison having been expelled before it could reach the tissues, which its contact would have disorganized. The venous, or absorbent system, is, therefore, that part of the vascular network which contributes to the production of general phenomena, when deleterious agents are introduced into the economy; nor do the arteries begin to play their part before the veins have accomplished theirs. But, as we have just stated, the poison is sometimes expelled before reaching the left side of the heart; and this elimination takes place on various points; but the pulmonary apparatus is the ordinary seat of the process. For this very reason the inner coat of the lungs is, perhaps, the most powerful of all absorbent surfaces; and poisons, when directly brought to bear upon it, produce a more instaneous effect than when deposited upon any other point; introduced, as it were, into the very centre of the arterial system, they pass at once into the circulation without any possibility of being expelled. Thus, sulphuretted hydrogen when introduced into the lungs, extinguishes life within a few seconds; while the same gas exists, to a considerable amount, in various parts of the intestinal tube, and even circulates in the veins, without producing the slightest inconvenience. It must, of course, be understood that the presence of poisons within the animal economy is perfectly inocuous as long as they are not collected together in sufficiently large quantities at a given moment. Small doses of the most dangerous substances may be successively poured into the vessels, at distant intervals, with perfect impunity. In estimating the noxious power of toxic bodies, the amount actually contained within the blood must alone be taken into consideration.—*Medical Times and Gazette.*

